# Supplementary material for: Maternal Selenium, Copper and Zinc Concentrations in Early Pregnancy, and the Association with Fertility
Source: Nutrients. 2019 Jul 16;11(7):1609. doi: 10.3390/nu11071609 (PMC6683068; doi:10.3390/nu11071609)
Supplement: Supplementary file 1 [file nutrients-11-01609-s001.pdf]

Supplementary Table S1: Association between plasma trace element concentrations measured at 15±1 weeks' gestation and time to pregnancy, excluding women requiring assisted reproductive technology

**Trace element**	**Concentration (µmol/L)**[a]	**n (%)**	**Unadjusted time ratio**	**Adjusted time ratio (95% CI)**		
				Model 1[b]	Model 2[c]	Model 3[d]
**Selenium**	<0.95	605	1.07 (0.95-1.22)	1.10 (0.97-1.25)	1.15 (0.99-1.33)	1.16 (0.99-1.36)
	≥0.95	410	1	1	1	1
**Zinc**	<7.80	223	1.19 (1.02-1.39)	1.19 (1.02-1.39)	1.18 (1.00-1.40)	1.21 (1.01-1.44)
	≥7.80 to ≤12.24	706	1	1	1	1
	>12.24	85	1.15 (0.92-1.45)	1.17 (0.93-1.47)	1.10 (0.83-1.48)	1.07 (0.79-1.44)
**Copper**	<25.97	236	1.12 (0.96-1.31)	1.12 (0.95-1.31)	1.11 (0.93-1.33)	1.12 (0.93-1.35)
	≥25.97 to ≤34.78	566	1	1	1	1
	>34.78	213	1.08 (0.92-1.27)	1.05 (0.89-1.24)	1.01 (0.83-1.24)	1.06 (0.86-1.30)

[a] Adjusted for maternal age, maternal body mass index, ethnicity, socioeconomic status, plasma C-reactive protein, pre-pregnancy alcohol consumption, pre-pregnancy smoking status, frequency of sexual intercourse prior to conception, multivitamin use in first trimester, and trace element of interest in multivitamin

[b] Adjusted for b plus intake of fast food, green leafy vegetables, fruit, and fish in the one month prior to conception

[c] Adjusted for c plus paternal age and paternal body mass index

Supplementary Table S2: Association between plasma trace element tertile measured at 15±1 weeks' gestation and time to pregnancy

Trace element	Concentration tertile (µmol/L)	n (%)	Unadjusted time ratio	Adjusted time ratio (95% CI)		
				Model 1[a]	Model 2[b]	Model 3[c]
**Selenium**	<0.86	449	0.99 (0.84-1.16)	0.96 (0.82-1.13)	1.01 (0.84-1.21)	1.02 (0.84-1.24)
	≥0.86 to ≤0.97	291	1	1	1	1
	>0.97	320	0.87 (0.73-1.03)	0.82 (0.69-0.98)	0.81 (0.66-0.99)	0.80 (0.65-0.99)
**Zinc**	<8.34	356	1.24 (1.06-1.46)	1.20 (1.02-1.41)	1.17 (0.98-1.41)	1.18 (0.98-1.43)
	≥8.34 to ≤9.91	350	1	1	1	1
	>9.91	353	1.02 (0.86-1.20)	1.01 (0.86-1.19)	0.94 (0.77-1.14)	0.93 (0.76-1.13)
**Copper**	<27.96	360	1.00 (0.85-1.18)	1.00 (0.85-1.18)	1.01 (0.84-1.22)	1.02 (0.84-1.24)
	≥27.96 to ≤32.59	347	1	1	1	1
	>32.59	353	0.98 (0.83-1.16)	0.96 (0.81-1.13)	0.97 (0.80-1.18)	1.02 (0.83-1.25)

[a] Adjusted for maternal age, maternal body mass index, ethnicity, socioeconomic status, plasma C-reactive protein, pre-pregnancy alcohol consumption, pre-pregnancy smoking status, frequency of sexual intercourse prior to conception, multivitamin use in first trimester, and trace element of interest in multivitamin

[b] Adjusted for b plus intake of fast food, green leafy vegetables, fruit, and fish in the one month prior to conception

[c] Adjusted for c plus paternal age and paternal body mass index

Supplementary Table S3: Risk for subfertility (>12 months to conceive) according to plasma trace element concentrations measured at 15±1 weeks' gestation, excluding women requiring assisted reproductive technology

Trace element	Concentration (μmol/L)[a]	N	n (%)	Unadjusted relative risk	Model 1[b]	Model 2[c]	Model 3[d]
**Selenium**	<0.95	605	84 (13.9)	1.54 (1.07-2.22)	1.59 (1.09-2.30)	1.58 (1.06-2.36)	1.60 (1.06-2.41)
	≥0.95	410	37 (9.0)	1	1	1	1
**Zinc**	<7.80	223	30 (13.5)	1.17 (0.79-1.73)	1.14 (0.77-1.69)	1.05 (0.70-1.59)	1.09 (0.72-1.65)
	≥7.80 to ≤12.24	706	81 (11.5)	1	1	1	1
	>12.24	85	10 (11.8)	1.03 (0.55-1.90)	1.11 (0.60-2.05)	0.95 (0.43-2.10)	0.78 (0.32-1.91)
**Copper**	<25.97	236	34 (14.4)	1.36 (0.92-2.01)	1.44 (0.96-2.16)	1.24 (0.82-1.88)	1.29 (0.84-1.98)
	≥25.97 to ≤34.78	566	60 (10.6)	1	1	1	1
	>34.78	213	27 (12.7)	1.20 (0.78-1.83)	1.07 (0.69-1.65)	0.96 (0.58-1.57)	1.04 (0.63-1.72)

[a] Adjusted for maternal age, maternal body mass index, ethnicity, socioeconomic status, plasma C-reactive protein, pre-pregnancy alcohol consumption, pre-pregnancy smoking status, frequency of sexual intercourse prior to conception, multivitamin use in first trimester, and trace element of interest in multivitamin

[b] Adjusted for b plus intake of fast food, green leafy vegetables, fruit, and fish in the one month prior to conception

[c] Adjusted for c plus paternal age and paternal body mass index

Supplementary Table S4: Risk for subfertility (>12 months to conceive) according to plasma trace element tertile measured at 15±1 weeks' gestation

**Trace element**	**Concentration tertile (μmol/L)**	**N**	**n (%)**	**Unadjusted relative risk**	**Model 1[a]**	**Model 2[b]**	**Model 3[c]**
**Selenium**	<0.86	449	79 (17.6)	1.02 (0.74-1.41)	0.97 (0.71-1.34)	1.02 (0.73-1.42)	1.05 (0.75-1.47)
	≥0.86 to ≤0.97	291	50 (17.2)	1	1	1	1
	>0.97	320	37 (11.6)	0.67 (0.45-1.00)	0.62 (0.42-0.91)	0.67 (0.44-1.02)	0.64 (0.41-1.00)
**Zinc**	<8.34	356	67 (18.8)	1.27 (0.91-1.76)	1.16 (0.84-1.62)	1.05 (0.75-1.47)	1.05 (0.75-1.49)
	≥8.34 to ≤9.91	350	52 (14.9)	1	1	1	1
	>9.91	353	47 (13.3)	0.90 (0.62-1.29)	0.89 (0.62-1.28)	0.79 (0.53-1.17)	0.73 (0.49-1.10)
**Copper**	<27.96	360	56 (15.6)	0.96 (0.69-1.35)	1.01 (0.72-1.43)	0.93 (0.65-1.33)	0.98 (0.68-1.41)
	≥27.96 to ≤32.59	347	56 (16.1)	1	1	1	1
	>32.59	353	54 (15.3)	0.95 (0.67-1.34)	0.88 (0.63-1.23)	0.93 (0.65-1.33)	1.01 (0.70-1.46)

[a] Adjusted for maternal age, maternal body mass index, ethnicity, socioeconomic status, plasma C-reactive protein, pre-pregnancy alcohol consumption, pre-pregnancy smoking status, frequency of sexual intercourse prior to conception, multivitamin use in first trimester, and trace element of interest in multivitamin
[b] Adjusted for b plus intake of fast food, green leafy vegetables, fruit, and fish in the one month prior to conception
[c] Adjusted for c plus paternal age and paternal body mass index
